# Paradoxical worsening of tuberculous chorioretinitis in a Chinese gentleman

**DOI:** 10.1186/s12348-015-0052-1

**Published:** 2015-07-09

**Authors:** Rosalynn Grace Siantar, Su Ling Ho, Rupesh Agrawal

**Affiliations:** Department of Ophthalmology, Tan Tock Seng Hospital, Singapore, 308433 Singapore

**Keywords:** Ocular tuberculosis, Chorioretinitis, Paradoxical worsening, Steroids

## Abstract

We report a case of paradoxical worsening of tuberculous chorioretinitis after initiation of anti-tuberculous therapy (ATT). The patient had left panuveitis with tuberculous chorioretinitis and was started on systemic ATT and oral steroids a week later. However, he developed paradoxical worsening 2 months after initiation of therapy. He was continued on ATT, oral steroids and intravitreal amikacin with resolution of the chorioretinal lesion subsequently. Ocular tuberculosis often poses a diagnostic challenge, and clinicians should be aware of the possibility of paradoxical worsening despite appropriate ATT. Clinicians should strongly consider starting oral steroids concurrently with ATT when managing ocular tuberculosis.

## Correspondence/findings

### Introduction

Tuberculosis (TB), caused by *Mycobacterium tuberculosis*, can cause multi-systemic granulomatous inflammation, most commonly in the pulmonary system. Although ocular involvement is relatively uncommon, it is still a well-known extrapulmonary manifestation [1]. It often poses a diagnostic challenge to ophthalmologists, and management should be prompt as it can sight saving. The commonest manifestation of ocular tuberculosis is in the uveal tract and usually presents as a posterior uveitis, of which choroidal tuberculomas are the commonest [1]. With anti-tuberculous therapy (ATT), posterior uveitis is expected to resolve within 4 to 6 weeks [1]. We report a rare case of paradoxical worsening of ocular TB after initiation of ATT.

### Report

**Fig. 1:**
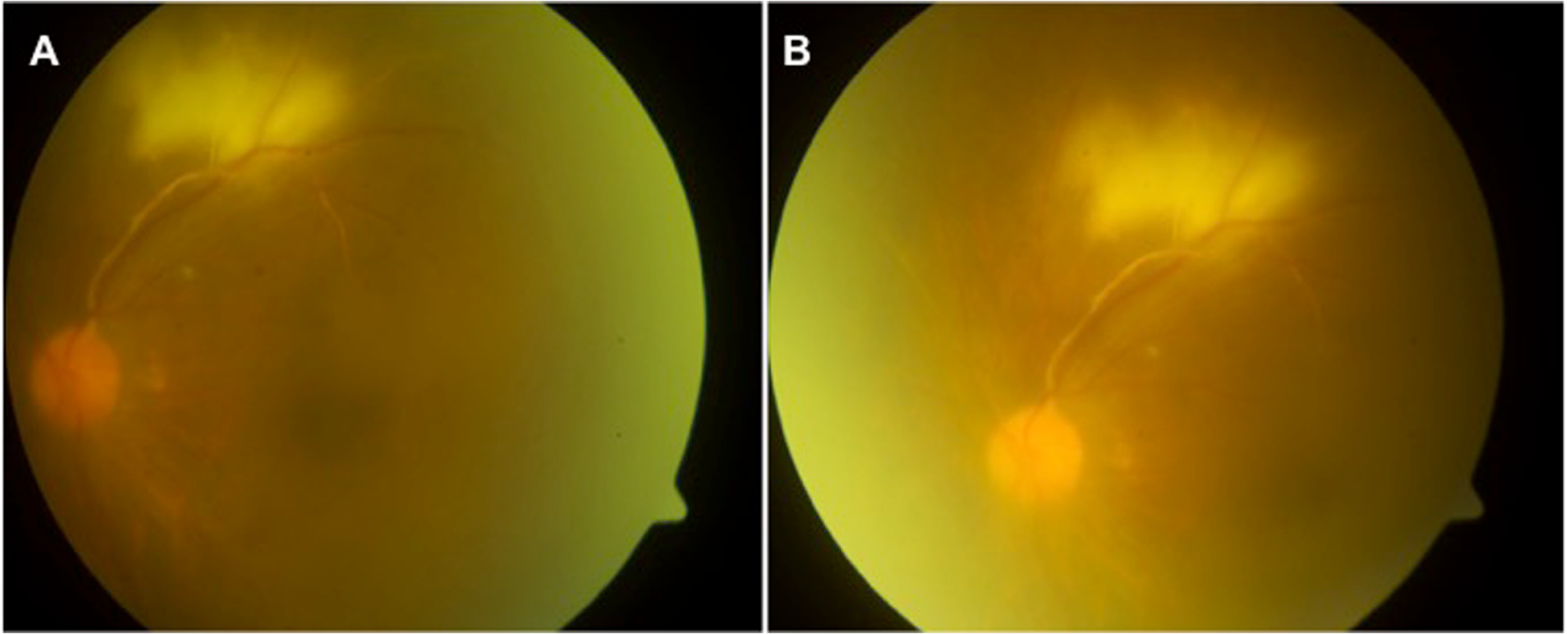
**a**, **b** Fundus photos of left eye showing vitritis, vasculitis and chorioretinitis superior to the macula

**Fig. 2:**
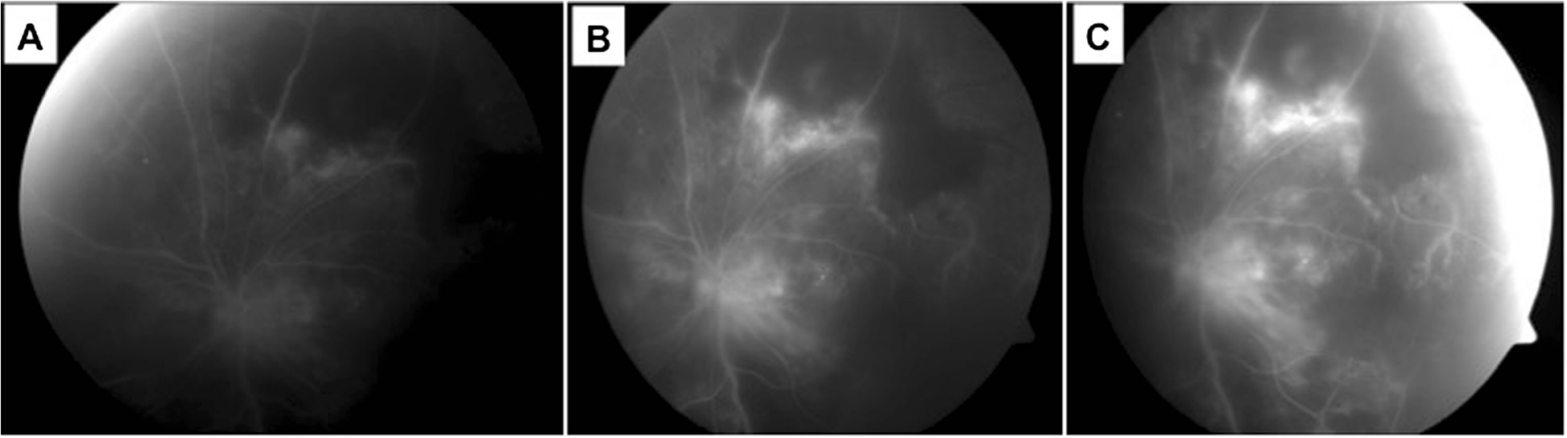
Fundus fluorescein angiography in early (**a**), mid (**b**) and late (**c**) phases showing non-perfusion peripheral to area of chorioretinitis

**Fig. 3:**
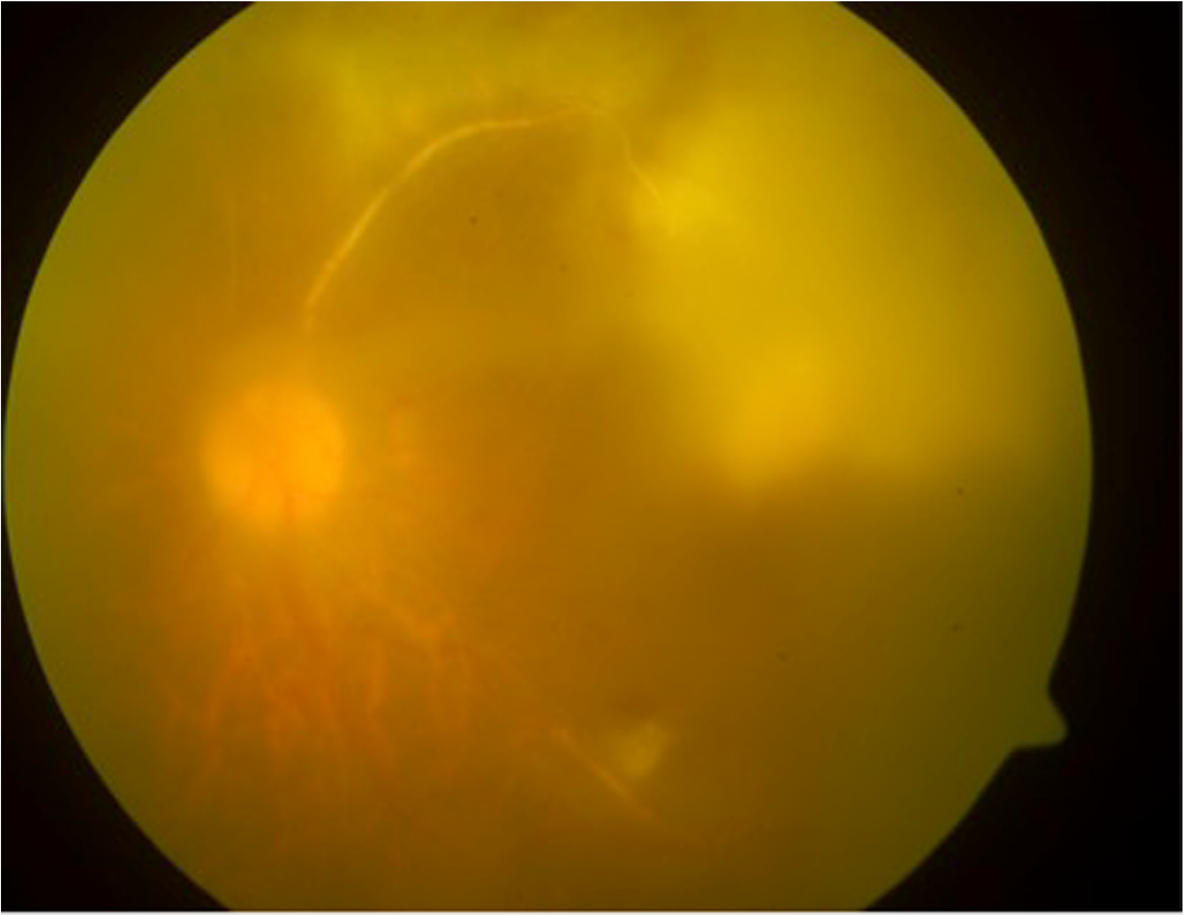
Fundus photo of the left eye showing progression of chorioretinitis to the macula

A 50-year-old man with past medical history of diabetes mellitus presented with 1 week history of left eye blurring of vision and mild discomfort. His Snellen visual acuity was 6/7.5 in the right eye and 6/15 in the left eye. Anterior segment examination revealed signs of uveitis with multiple keratic precipitates, anterior chamber cells 1+ and retrolental cells 2+. Fundus examination showed significant vitritis, vasculitis and a well defined yellowish-white chorioretinal lesion superior to the macula (Fig. 1a, b). Anterior and posterior segment examination of the right eye was normal. He underwent a vitreous tap which identified mycobacterial DNA via polymerase chain reaction (PCR). The PCR test was the Probetec DTB assay (Becton Dickinson), and the gene sequences targeted were IS6110 and 16S rRNA. His Mantoux test measured 25 mm and chest X-ray was clear. Full blood count revealed lymphocytosis, and other systemic investigations including HIV and syphilis were negative. The working diagnosis was that of a left eye panuveitis with tuberculous chorioretinitis, and he was started on ATT (6 months course of oral rifampicin, isoniazid and ethambutol) by the infectious disease specialist. He was subsequently started on oral prednisolone 40 mg a week later. Fundus fluorescein angiogram (Fig. 2a–c) revealed non-perfusion peripheral to the choroidal lesion representing a branch retinal vein occlusion, and he underwent a sectoral panretinal photocoagulation. In the third month of treatment, the patient was noted to have clinical deterioration with decrease in visual acuity to hand movement and progression of the chorioretinal lesion to the macula (Fig. 3). He underwent a repeat vitreous tap for which mycobacterial DNA PCR was now negative and received a dose of intravitreal amikacin 0.4 mg/0.1 ml. The patient was continued on systemic oral ATT and steroids, and the lesion slowly resolved, becoming smaller in size and developing fibrosis and later scarring. The patient’s final visual acuity at last follow-up was 6/120. The patient has given consent for the report to be published.

## Discussion

In systemic tuberculosis, paradoxical worsening has been described as worsening of intracranial tuberculoma, meningeal disease, tuberculous meningeal radiculitis, pleural effusion and abdominal tuberculosis [1]. Paradoxical worsening after antibiotic therapy has been described in other conditions and is termed Jarisch-Herxheimer reaction (JHR) when associated with the treatment of secondary syphilis, manifesting with systemic symptoms such as fever, headache and sweating [2].

Isolated intraocular JHR has been previously reported in treatment of ocular syphilis with rapid visual loss [3]. Worsening after initial therapy has also been described in other conditions such as Whipple’s disease [4] and Lyme disease [5].

**Fig. 4:**
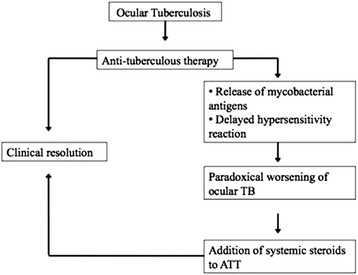
Diagrammatic representation of clinical course of ocular tuberculosis

The pathogenesis of paradoxical worsening in tuberculosis is not well understood. Proposed mechanisms include release of mycobacterial antigens after ATT and delayed hypersensitivity [1]. It can clinically manifest as rapid worsening of vitritis and chorioretinitis and has been reported to have good response to the addition of oral steroid [6–8] (Fig. 4).

In a case series published by Hamade et al. [9], 20 patients with presumed ocular tuberculosis treated with ATT only had complete resolution with no complication. On the other hand, although paradoxical worsening of ocular tuberculosis after treatment with ATT is rare, there have been a few cases reported so far [6–8, 10]. Interestingly, in a case series of 110 patients published by Gupta et al. [11], 14 % of patients with tubercular serpiginous-like choroiditis had continued progression while on treatment. One patient was on corticosteroids only, while the rest were on ATT and corticosteroids when they were observed to have continued progression. They were managed with increased immunosuppression either with increased dosage of corticosteroids or other immunosuppressants such as azathioprine, and these patients subsequently achieved clinical resolution. These studies demonstrate variability in response of ocular TB to different treatment regimes and highlight a need for future studies with a larger population of ocular tuberculosis to better evaluate the ideal treatment regime of ATT with or without adjunctive systemic steroids.

Our patient developed paradoxical worsening despite already being on oral steroid and ATT. In our case, oral steroid was initiated 1 week after the initiation of ATT. This is unique among other reported cases where steroid was only added to the treatment regime after development of ocular paradoxical worsening or started concurrently with ATT [11]. It is important to highlight that rifampicin, which is usually part of the standard regimen of ATT, may reduce bioavailability of prednisolone by 66 % [12]. This should be taken into consideration in order to achieve the therapeutic dose of systemic steroids in such patients. The benefit of starting oral steroids concurrently with ATT remains to be seen and needs further studies.

## Conclusion

Ocular tuberculosis often poses a diagnostic and management challenge. Clinicians should be aware of the possibility of paradoxical worsening of ocular tuberculosis despite initiating ATT and consider starting systemic oral steroids concurrently and at the right dosage when managing ocular tuberculosis.

## Abbreviations

ATT: anti-tubercular therapy
DNA: deoxyribonucleic acid
HIV: human immunodeficiency virus
JHR: Jarisch-Herxheimer reaction
PCR: polymerase chain reaction
TB: tuberculosis
